# Increased Adult *Aedes aegypti* and *Culex quinquefasciatus* (Diptera: Culicidae) Abundance in a Dengue Transmission Hotspot, Compared to a Coldspot, within Kaohsiung City, Taiwan

**DOI:** 10.3390/insects9030098

**Published:** 2018-08-13

**Authors:** Ka-Chon Ng, Luis Fernando Chaves, Kun-Hsien Tsai, Ting-Wu Chuang

**Affiliations:** 1 College of Public Health, National Taiwan University, Taipei 10055, Taiwan; chonchonedwin0719@gmail.com (K.-C.N.); kunhtsai@ntu.edu.tw (K.-H.T.); 2 Instituto Costarricense de Investigación y Enseñanza en Nutrición y Salud (INCIENSA), Apartado Postal 4-2250, Tres Ríos, Cartago, Costa Rica; lfchavs@gmail.com; 3 Programa de Investigación en Enfermedades Tropicales (PIET), Escuela de Medicina Veterinaria, Universidad Nacional, Apartado Postal 304-3000, Heredia, Costa Rica; 4 Department of Molecular Parasitology and Tropical Diseases, School of Medicine, College of Medicine, Taipei Medical University, No. 250, Wuxing Street, Xinyi District, Taipei 11031, Taiwan

**Keywords:** Schmalhausen’s law, time series analysis, pest outbreaks, kurtosis, weather variability

## Abstract

The assumption that vector abundance differences might drive spatial and temporal heterogeneities in vector-borne disease transmission is common, though data supporting it is scarce. Here, we present data from two common mosquito species *Aedes aegypti* (Linnaeus) and *Culex quinquefasciatus* Say, biweekly sampled as adults, from March 2016 through December 2017, with BG-sentinel traps in two neighboring districts of Kaohsiung City (KC), Taiwan. One district has historically been a dengue transmission hotspot (Sanmin), and the other a coldspot (Nanzih). We collected a total 41,027 mosquitoes, and we found that average mosquito abundance (mean ± S.D.) was higher in Sanmin (*Ae. aegypti*: 9.03 ± 1.46; *Cx. quinquefasciatus*: 142.57 ± 14.38) than Nanzih (*Ae. aegypti*: 6.21 ± 0.47; *Cx. quinquefasciatus*: 63.37 ± 8.71) during the study period. In both districts, *Ae. aegypti* and *Cx. quinquefasciatus* population dynamics were sensitive to changes in temperature, the most platykurtic environmental variable at KC during the study period, a pattern predicted by Schmalhausen’s law, which states that organisms are more sensitive to small changes in environmental variables whose average value is more uncertain than its extremes. Our results also suggest that differences in *Ae. aegypti* abundance might be responsible for spatial differences in dengue transmission at KC. Our comparative approach, where we also observed a significant increment in the abundance of *Cx. quinquefasciatus* in the dengue transmission hotspot, suggests this area might be more likely to experience outbreaks of other vector borne diseases and should become a primary focus for vector surveillance and control.

## 1. Introduction

The yellow fever mosquito, *Aedes aegypti* (Linnaeus), and the southern house mosquito, *Culex quinquefasciatus* Say, are two of the most common urban mosquitoes in tropical and subtropical environments around the world [1,2,3]. *Ae. aegypti* is the main vector of dengue, chikungunya, and Zika in subtropical and tropical regions [4,5,6], while *Cx. quinquefasciatus* is a vector of worms causing lymphatic filariasis [7], West Nile Virus, WNV [8], and St Louis Encephalitis Virus [9], and both species are major causes of nuisance to humans [10]. *Cx. quinquesfaciatus* is one of the few species where impacts of global warming on its overwintering patterns have been observed in subtropical environments [11], while *Ae. aegypti* is a species whose distribution seems to be expanding as a result of global warming [12,13,14]. With changing climatic patterns, increased movement of people and goods, and unplanned urbanization, the transmission risk of vector-borne diseases has seen an unprecedented growth across the globe [15,16,17]. Dengue fever is one of the vector-borne diseases that has seen a significant worldwide geographical expansion [18,19] and the most important vector-borne disease in Taiwan [20].

Mosquito abundance is a key parameter for the infection risk of vector-borne diseases [21,22,23]. Various climatic factors impact mosquito ecology and pathogen development and transmission [24,25]. An ubiquitous pattern is that high temperatures reduce mosquito developmental time [26,27,28,29] and also reduce the extrinsic incubation period of pathogens—for example, dengue virus in *Ae. aegypti* [30] and WNV in *Culex* spp. mosquitoes [8,31]. Depending in the environmental context, high temperatures can also drive mosquito abundance outbreaks, i.e., sudden increases in the abundance of an insect [32,33]. Relative humidity might influence the survival and flight ability of vectors [34,35,36]. The effect of precipitation, by contrast, depends on the environmental context where mosquitoes live [24]. For example, in dry environments moderate rainfall is required to create more habitats for vectors [37], rainfall can drive vector abundance outbreaks in wet environments [38,39], and rainfall can drive the recruitment of larvae from diapausing eggs [40]. However, rainfall impacts are highly nonlinear [39], given that extreme rainfall might wash-out larvae from aquatic habitats depending on the mosquito species [41]. In addition, droughts might trigger water accumulation in artificial containers by humans, which can increase the recruitment of adult mosquito vectors [42,43,44].

Although no single mosquito sampling method can capture all mosquito species in a given area [45,46,47] nor satisfactorily allow the estimation of mosquito population size [48], systematic sampling with a single method could help to understand the impact of environmental fluctuations on mosquito species abundance [24], and vector infection at a given place [49,50]. Moreover, insights about mosquito population dynamics [51,52,53] and pathogen transmission [54,55] become increasingly robust as observations are not restricted to a single season. In addition, systematic sampling could also help to understand differences in the entomological risk for disease transmission over space and time [50,55,56].

Kaohsiung City (KC) has been the epidemic center of dengue outbreaks in 2014 (14,971 confirmed cases) and 2015 (19,769 confirmed cases) in southern Taiwan [57,58,59]. Our previous work in southern Taiwan demonstrated important associations between both regional and local climatic fluctuations on dengue transmission [20] and spatio-temporal analyses of dengue incidence has shown the existence of persistent dengue transmission hotspots and coldspots in KC [60,61,62]. One hypothesis for spatial heterogeneity in dengue transmission is that hot and cold spots are associated with different patterns of mosquito abundance and response to environmental fluctuations, which can be specific to dengue vectors or shared with other mosquito species commonly co-occurring with dengue vectors. To test this hypothesis, we biweekly collected mosquitoes during 2016 and 2017 with BG-sentinel traps [63], placed in two highly urbanized districts of KC, one part of a dengue transmission hotspot and one of a dengue transmission coldspot. We used time series analysis to evaluate differences in the association between vector abundance and weather fluctuations in *Ae. aegypti*, the dominant dengue vector in southern Taiwan, and *Cx. quinquefasciatus*, a common urban nuisance mosquito, and a species also competent to transmit several pathogens.

## 2. Materials and Methods

### 2.1. Study Area and Weather Data

**Figure 1:**
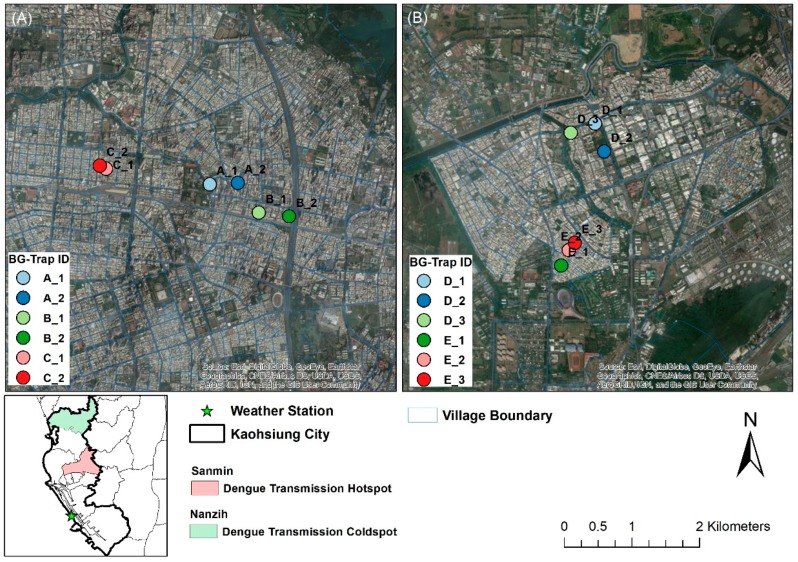
Study Locations. (**A**) Sanmin—dengue transmission hotspot (**B**) Nanzih—dengue transmission coldspot. The small map shows the location of Kaohsiung City, southern Taiwan, and also Sanmin and Nanzih districts.

Kaohsiung City (KC) is a city with 2.8 million habitants and a density of 942 people/km^2^ (Department of Household Registration, Ministry of Interior). KC is the main dengue epidemic center in Taiwan and accounts for more than 70% of dengue cases in the whole country (Taiwan Centers for Disease Control). Two highly urbanized (i.e., with over 40% of land cover used for housing and roads) districts of KC—Sanmin and Nanzih—were selected for the study. Sanmin district has historically been a dengue transmission hotspot with a high annual incidence rate (209.5 per 100,000). In contrast, Nanzih has been a dengue transmission coldspot, where dengue incidence rate is about 47.9 per 100,000. The location of the districts and sampling locations within each district are shown in Figure 1, and coordinates and pictures of the places where mosquito traps were located are presented in Supplementary Table S1 [online only]. For each sampling location, we evaluated the percent land cover in a circular buffer of 500 m radius around each trap, the maximum recorded dispersal distance of *Ae. aegypti* in urban landscapes [64], and a radius were most host seeking *Cx. quinquefasciatus* occur in relation to their larval habitats of origin [65]. The percent of urbanized land surrounding the traps was (mean ± S.D.): 55.75 ± 5.32 in Sanmin and 49.61 ± 8.49 in Nanzih, values not significantly different according to a Welch’s *t* test (*t* = 1.501, df = 8.408, *p*-value = 0.17). Moreover, landscape configuration, assessed through a Principal Components Analysis (PCA) [65,66] of the percent of land used for housing, roads, parks, and other purposes, described in Supplementary Figure S1 [online only], also showed no significant statistical differences between the two sites (*t* = 2.314, df = 7.195, *p*-value = 0.05289) when compared with a Welch’s *t* test, a *t* test that corrects df considering variance differences between the groups compared [67]. Despite the similarities in land cover, the studied districts have different population density per square KM, with Sanmin (17,321 habitants/KM^2^) more than doubling the population density of Nanzih (7130 habitants/KM^2^).

**Figure 2:**
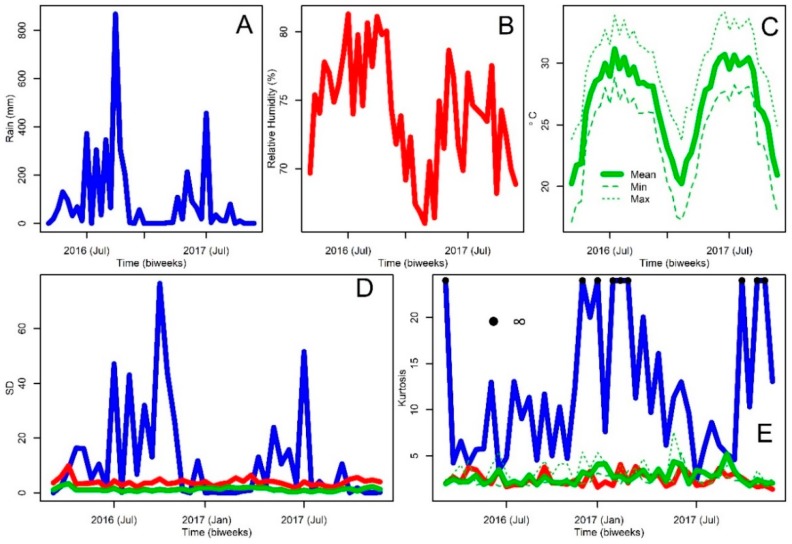
Weather Time series data at Kaohsiung, Taiwan. (**A**) Rainfall in mm; (**B**) Relative humidity, %; (**C**) Temperature including Maximum (Max), Minimum (Min), and Average (Mean), see inset legend for details about the lines. (**D**) Standard deviation, SD, of the environmental variables, lines are color coded according to panels A, B, and C. (**E**) Kurtosis of the environmental variables, lines are color coded according to panels A, B, and C, for rainfall kurtosis black dots indicate infinite values, a perfect leptokurtic distribution estimated during periods without rainfall.

Only one weather station (Kaohsiung Weather Service Office; ID:46744 (120.30° E, 22.57° N)) (Figure 1) exists within the limits of KC, so all the climate variables used in this analysis, including daily average temperature, daily average precipitation, and daily average relative humidity, were summarized from the hourly measurements of the weather station. The study period coincided with one of the strongest El Niño events on record [66,67], and as consequence of this phenomenon, weather patterns were anomalous in KC. During the last quarter of 2016, precipitation was higher than usual in KC (Supplementary Figure S2A [Online Only]). Meanwhile, mean temperature (Supplementary Figure S2B [Online Only]) was hotter than usual during the whole study period, a pattern also observed for maximum (Supplementary Figure S2C [Online Only]) and minimum temperature (Supplementary Figure S2D [Online Only]). For the analysis, we employed daily data from 1 January 2016 to 31 December 2017, which was processed at biweekly intervals coinciding with mosquito sampling. For each time interval, we computed average values and the standard deviation and kurtosis of the above mentioned weather variables to understand the impacts of environmental variability on mosquito population dynamics [40]. The average distance between the weather station and mosquito traps was 12.13 KM (SD: 3.96 KM, Range: 8.1–16.9 KM). The resulting biweekly weather data can be observed in Figure 2. In Kaohsiung City, winters, from December to February, have low rainfall (Figure 2A), low relative humidity (Figure 2B), and temperature also drops to a minimum (Figure 2C). By contrast, during the summer, i.e., June to August, rainfall (Figure 2A), relative humidity (Figure 2B), and temperature (Figure 2C) reach their maximum values. With the exception of rainfall, which had a strong variation and which was followed in magnitude by relative humidity, the SD of all temperature variables was similar and low during the study period (Figure 2D). Meanwhile, the most leptokurtic variable, i.e., with high kurtosis values which represent low variation around the mean [68], where rainfall and maximum temperature, which reached maximum values from November to March, while the most platykurtic variable, more uncertain around the mean value than the extremes of its distribution [69], was minimum temperature (Figure 2E).

### 2.2. Mosquito Sampling

In this study, we collected mosquitoes with a total of 12 BG-Sentinel traps (Biogents, A.G. Regensburg, Germany). Traps were set in the two small area districts of Kaohsiung City: Sanmin (19.79 KM^2^) and Nanzih (25.83 KM^2^), as shown in Figure 1. Six traps were placed in each district, from 3 March 2016 to 26 December 2017, a number constrained by our limited budget and manpower but appropriate for the small area districts that we studied. Traps were baited with BG lure, a substance that mimics compounds released by the human skin [70]. Baits were replaced every two months following manufacturer recommendations [63,71]. Traps were operated continuously and mosquito samples were collected by laboratory staff on a bi-weekly basis. All traps were set in sheltered open household spaces, e.g., yards and/or garages, and household owners kindly provided electricity for trap operation. BG traps baited with BG lure were primarily chosen given their ability to capture *Aedes* (*Stegomyia*) spp., for example, *Ae. aegypti* and *Aedes albopictus* (Skuse) [62,72], but BG traps are known to capture a diverse set of mosquito species, including *Culex* (*Culex*) spp. [62,73,74] with medical importance such as *Cx. quinquefasciatus* in subtropical environments [63,73]. At the landscape level, all traps were located in households with similar environmental characteristics, in urban areas dominated by residential use. Mosquito samples were collected by the laboratory staff and mosquitoes were identified according to morphological characteristics presented in the taxonomic key by JC Lien [75]. Both *Aedes* spp. and *Culex* spp. were included in the analysis. Only female mosquitoes were considered in the study due to their importance for disease transmission.

### 2.3. Statistical Analysis

We analyzed average time series for Sanmin (dengue transmission hotspot) and Nanzih (dengue transmission coldspot) districts of KC. We analyzed average time series because a couple of the traps broke during the first year of the study, which we could not replace because of limited funding. When estimating the biweekly mean abundance, we considered all traps available each week, since there was no significant difference on the estimated mean abundance for each mosquito species and study location when these traps were excluded (Appendix A), thus ensuring mean estimates were not biased by including data from the traps that broke at the start of the study. For the time series analysis, we employed autoregressive (AR) models [76]. On seven occasions, it was not possible to collect samples on the exact date scheduled for sampling. Therefore, before the time series analysis, we used the lowess method to interpolate observations for the missing dates [77] in order to fulfill the assumption of data sampled at equal time intervals of AR models [76]. To fit the AR time series models, we followed a protocol described in detail by T Hoshi, Y Higa and LF Chaves [78], where null AR models are fit to mosquito abundance time series after selecting lags by inspecting the autocorrelation function (ACF) and then confirmed by inspecting the partial autocorrelation function (PACF) of the time series. Then, the time series of environmental covariates are pre-whitened using the coefficients of the null AR models. The resulting time series are used to estimate cross correlation functions (CCF), which are employed to select environmental covariates and their lags. The selected variables are then included in a full model for each mosquito time series, which is then simplified by a process of backward elimination based on the minimization of the Akaike Information Criterion (AIC). Prior to fitting each time series model, the mean of each environmental variable is removed in order to ease the interpretation of the time series model intercept as the mean value of the time series [23]. For further details about the methods, please refer to T Hoshi, Y Higa and LF Chaves [78] and LF Chaves, N Imanishi, and T Hoshi [23].

## 3. Results

**Figure 3:**
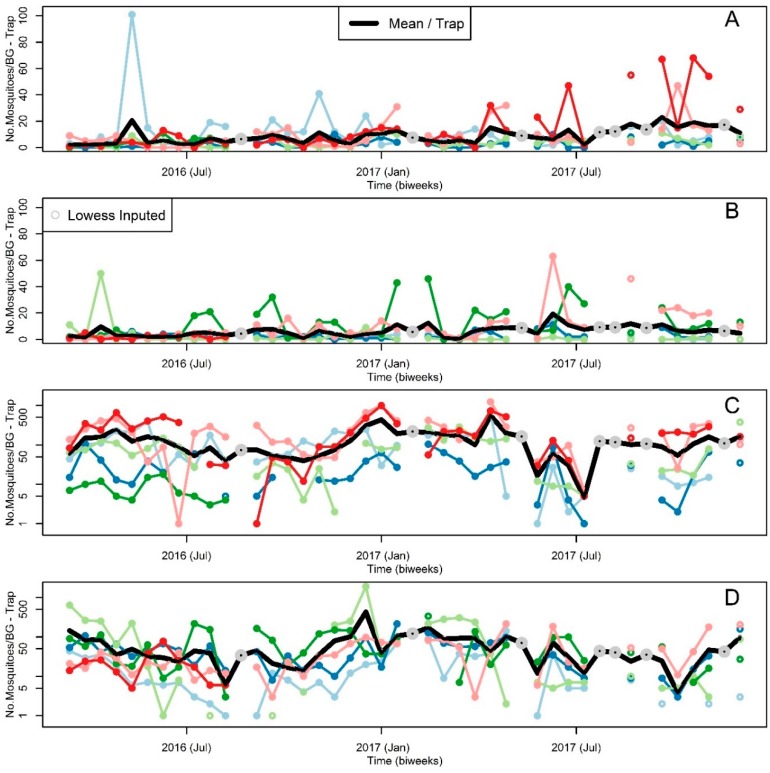
Mosquito Abundance Time Series. *Aedes aegypti* per BG Sentinel trap collection in (**A**) Sanmin and (**B**) Nanzih. *Culex quinquefasciatus* per BG Sentinel trap collection in (**C**) Sanmin and (**D**) Nanzih. At each site line colors correspond to the trap locations in Figure 1, with the exception of black lines which represent average values, see inset legend in (**A**). Grey circles indicate abundance estimates interpolated with lowess, see inset legend in (**B**). To ease visualization the y axis in (**C**,**D**) is shown in logarithmic scale.

We collected a total of 41,027 mosquitoes, over a total 746 biweekly trap collections. We collected a total of eight *Aedes albopictus* (Skuse), 2729 *Ae. Aegypti*, and 38,290 *Cx. quinquefasciatus*. In each one of the districts, we collected 4 *Ae. albopictus* during the full study period*.* In contrast, the average (±S.D.) abundance per trap and biweek of *Ae. aegypti* (9.03 ± 1.46) and *Cx. quinquefasciatus* (142.57 ± 14.38) were higher in Sanmin (Figure 3A,C) than Nanzih (*Ae. aegypti*: 6.21 ± 0.47 see Figure 3B; *Cx. quinquefasciatus*: 63.37 ± 8.71 see Figure 3D). Figure 3 also shows that starting in 26 August 2016 we lost two of the traps, reducing the number of traps to five at each site. Overall, the population dynamics of *Ae. aegypti* at Sanmin (Figure 3A) and Nanzih (Figure 3B) did not show major fluctuations, on average, through the study period. Nevertheless, some locations showed “outbreaks” in the sense, that often, sudden extraordinary changes in the abundance of *Ae. aegypti* were observed, which were larger in Sanmin (Figure 3A) when compared to Nanzih (Figure 3B).

**Figure 4:**
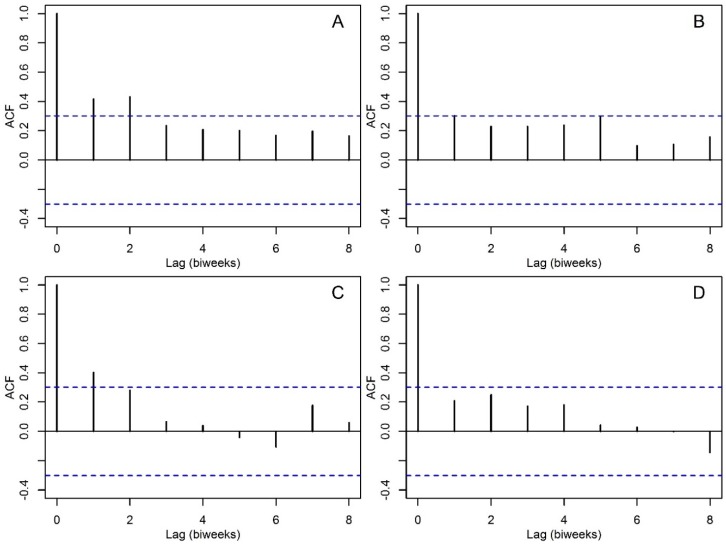
Autocorrelation functions (ACF) *Aedes aegypti* per BG Sentinel trap collection in (**A**) Sanmin and (**B**) Nanzih. *Culex quinquefasciatus* per BG Sentinel trap collection in (**C**) Sanmin and (**D**) Nanzih. In the panels, dashed blue lines are 95% confidence limits within which correlations are expected to occur by chance.

In general, all time series were autocorrelated (Figure 4), and patterns of autocorrelation were mainly suggestive of first order AR processes, i.e., significant autocorrelation at one time lag, for *Ae. aegypti* in Sanmin (Figure 4A) and Nanzih (Figure 4B) and *Cx. quinquefasciatus* in Sanmin (Figure 4C) but not in Nanzih (Figure 4D). Using this information, first order autoregressive models were fitted as null models to all time series but *Cx. quinquefasciatus* at Nanzih (Figure 4D).

**Figure 5:**
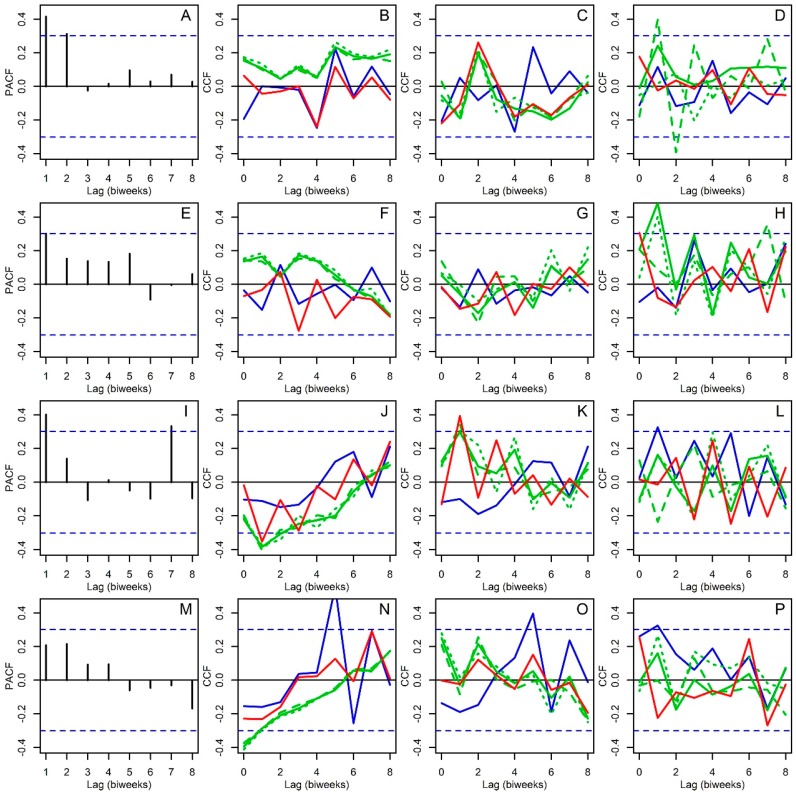
Partial autocorrelation (PACF) and cross correlation functions (CCF). *Aedes aegypti* in Sanmin: (**A**) PACF and (**B**) CCF with mean values of environmental variables (**C**) CCF with SD values of environmental variables (**D**) CCF with kurtosis values of environmental variables. *Ae. aegypti* in Nanzih: (**E**) PACF and (**F**) CCF with mean values of environmental variables (**G**) CCF with SD values of environmental variables (**H**) CCF with kurtosis values of environmental variables. *Culex quinquefasciatus* in Sanmin: (**I**) PACF and (**J**) CCF with mean values of environmental variables (**K**) CCF with SD values of environmental variables (**L**) CCF with kurtosis values of environmental variables. *Cx. quinquefascuatus* in Nanzih: (**M**) PACF and (**N**) CCF with mean values of environmental variables (**O**) CCF with SD values of environmental variables (**P**) CCF with kurtosis values of environmental variables. In all panels dashed blue lines are 95% confidence limits within which correlations are expected to occur by chance. In the CCF plots lines, environmental variables are color coded following the pattern described in Figure 3.

The PACF of the *Ae. aegypti* time series from Sanmin furtherly suggested a first order AR process (Figure 5A). This time series was not associated with the mean (Figure 5B) and SD (Figure 5C) of any weather variable, but it was associated positively at lag 1 and negatively at lag 2 with minimum temperature kurtosis (Figure 5D). By contrast, the PACF of *Ae. aegypti* in Nanzih did not show any significant autoregression (Figure 5E) and like the pattern observed in Sanmin the Nanzih time series was not correlated the mean (Figure 5F) and SD (Figure 5G) of any weather variable. However, it was associated positively at lag 1 with the minimum, mean and maximum temperature kurtosis (Figure 5H). Meanwhile, the PACF of the *Cx. quinquefasciatus* time series from Sanmin confirmed a first order AR process, with a significant correlation at lag 7 that was ignored when fitting the time series models (Figure 5I). This time series was significantly associated at lag 1 with the mean (Figure 5J) and SD (Figure 5K) of temperature (mean, maximum and minimum) and relative humidity and rainfall kurtosis (Figure 5L). The PACF of *Cx. quinquefasciatus* at Nanzih, by contrast, showed no significant correlation (Figure 5M) and was associated with the mean (Figure 5N) and SD (Figure 5O) of rainfall with five biweeks of lag, with temperature (minimum, mean and maximum) at lag 0 (Figure 5N), and one biweek of lag with rainfall kurtosis (Figure 5P).

Using the information from Figure 5, we then fitted full models, including all significantly correlated lags of environmental time series, for each mosquito time series. These full models were simplified by backward elimination based on the minimization of the AIC, and each process of model selection is presented as Supplementary online materials: *Ae. aegypti* at Sanmin (Supplementary Table S2 [online only]) and Nanzih (Supplementary Table S3 [online only]); *Cx. quinquefasciatus* at Sanmin (Supplementary Table S4 [online only]) and Nanzih (Supplementary Table S5 [online only]). Parameter estimates for the best time series models for *Ae. aegypti* are presented in Table 1.

The biweekly average number (Intercept in Table 1) of *Ae. aegypti* mosquitoes was 50% higher in Sanmin (around 9) when compared with Nanzih (around 6) and in both locations the dynamics were sensitive to changes in minimum and mean temperature kurtosis, which were the more platykurtic environmental variables at KC during the study period (Figure 2E). Parameter estimates for the best time series models for *Cx. quinquefasciatus* are presented in Table 2. *Cx. quinquefasciatus* average abundance at Sanmin (around 143) more than doubled what we found at Nanzih (around 63). In both districts increasing temperature had a negative impact on *Cx. quinquefasciatus* abundance, with a larger coefficient in Sanmin (Table 2), but only at Nanzih did the SD of Rainfall had a positive impact (Table 2) on *Cx. quinquefasciatus* abundance.

## 4. Discussion

Our data shows that, during the study period (March 2016–December 2017), a dengue transmission hotspot consistently had more mosquitoes than a neighboring transmission coldspot at KC, Taiwan. This fact supports the hypothesis that differences in transmission between these two districts might be related to differences in vector abundance. Moreover, the fact that both *Ae. aegypti* and *Cx. quinquefasciatus* were more abundant in the hotspot than the coldspot highlights the need to focus vector surveillance and control in hotspot areas. Specific to dengue transmission ecology in KC, our data showed the presence of *Ae. aegypti* near households, an observation furtherly supporting that *Ae. aegypti* might play the most important role in dengue virus transmission in southern Taiwan [79,80].

Our longitudinal mosquito data also highlights the importance of high order environmental variability, measured by kurtosis, on forcing mosquito abundance fluctuations, a phenomenon observed elsewhere for *Ae. aegypti* [32,39], *Cx. quinquefasciatus* [81,82,83] and other mosquito species [23,38,71,84], with patterns following the prediction of Schmalhausen’s law, the biological principle stating that organisms are sensitive not only to average environmental conditions but also to the environmental variability *per se* [25]. In our study, both *Ae. aegypti* and *Cx. quinquefasciatus* were sensitive to temperature, the most platykurtic environmental variable during the study period, yet *Ae. aegypti* fluctuations were associated with changes in temperature kurtosis, while *Cx. quinquefasciatus* tracked changes in maximum temperature. These differences might be related to different impacts of temperature on the life history of these two mosquito species. For example, *Ae. aegypti* abundance changes might reflect an accelerated larval development to avoid death by extreme hot temperatures, and/or by larval habitat desiccation [32,39,85]. Meanwhile, abundance changes in *Cx. quinquefasciatus* could be related to decreased oviposition [86,87] and reduced aquatic survival at extreme high temperatures [88]. The different feeding behaviors of *Ae. aegypti* (day time biter) and *Cx. quinquefasciatus* (night time biter) might also be associated with the environmental conditions at their active time [10,89].

Although our longitudinal study was long enough to show the importance of platykurtic environmental factors on mosquito abundance fluctuations, it is an open question if impacts of the El Niño Southern Oscillation (ENSO) on dengue transmission [20,90,91,92] could be related to changes in vector abundance. For example, in leishmaniasis we have shown that vector abundance and transmission increase during the cold ENSO phase in Panamá [93,94]. One limitation of this study is that given the lack of baseline longitudinal studies it is impossible to assess if ENSO impacted the population dynamics of both *Ae. aegypti* and *Cx. quinquefasciatus*, especially considering this study was done during one of the strongest ENSO events on record [66,67], which altered weather patterns at KC (Supplementary Figure S2 [Online Only]), and also altered weather and phenological patterns of mosquitoes elsewhere in east Asia, as documented for *Tripteroides bambusa* (Yamada) in Nagasaki, Japan, where the comparison of records from the 1970’s and 1980’s with data from 2016 and 2017 showed an increase in winter temperatures and that pupal recruitment season has lengthened its duration [95].

One potential limitation of this study was the use of weather data from KC’s weather station, which was around 12 km apart from the study sites. Our previous experience has been that over spatial scales similar to the one of this study [68,95], and even larger [71] temperature fluctuations are more synchronous than mosquito abundance, and very similar to observations from weather stations located at a distance similar to the one between KC’s weather station and our study locations [95]. Based on that, we believe data from KC’s weather station was appropriate to evaluate the association between weather fluctuations and mosquito abundance at the two study sites. Nevertheless, it is an open question whether more finely grained weather data, from weather sensors attached to the traps, could help to improve the understanding of weather impacts on vector abundance at finer spatial scales.

Finally, our findings, in conjunction with observations about the high correlation between entomological indicators and dengue transmission [54], support the need to strengthen systematic vector surveillance at sites that consistently behave as dengue transmission hotspots and the importance of mosquito abundance for transmission, since we significantly found more mosquitoes in Sanmin (the dengue transmission hotspot) than in Nanzih (the dengue transmission coldspot), though these patterns might also be affected by other environmental, demographic, and social aspects important for vector-borne disease transmission [24,25]. In that sense, the development of intelligent mosquito traps that automatically count and identify mosquitoes is a very promising tool that could increase the reliability of early warning systems for vector-borne disease transmission, which primarily rely on weather data [96,97] given delays associated with the processing of entomological information [49,98].

## 5. Conclusions

Our data suggest that spatially heterogeneous *Ae. aegypti* abundance might be a driver of spatial differences in dengue transmission at KC. Our comparative approach, where we also observed a significant increment in the abundance of *Cx. quinquefasciatus* in the dengue transmission hotspot, suggests this area might be more likely to experience outbreaks of other vector borne diseases and should become a primary focus for vector ecology research, surveillance and control.

## Figures and Tables

**Table 1:** Parameter estimates for the best time series explaining abundance changes in *Aedes aegypti* sampled with BG-sentinel traps at Sanmin (dengue transmission hotsop) and Nanzih (dengue transmission coldspot), Kaohsiung City, Taiwan. In parameters, Intercept is the mean value of the time series and AR = autoregressive term, K = kurtosis, T = temperature, mean = average and min = minimum. Lags are in biweeks.

Parameters (lag)	Sanmin				Nanzih			
	Estimates	S.E.	z	*p*-Value	Estimates	S.E.	z	*p*-Value
Intercept	9.0337	1.4571	6.19	6.65 × 10^−10^ *	6.2149	0.4716	13.18	1.18 × 10^−39^ *
AR (1)	0.5537	0.1287	4.30	1.69 × 10^−5^ *	-	-	-	-
KTmin (1)	2.0087	0.8928	2.25	0.024 *	-	-	-	-
KTmin (2)	−2.0886	0.8976	−2.33	0.019 *	-	-	-	-
KTmean (1)	-	-	-	-	2.6512	0.591	4.49	7.26 × 10^−6^ *
Error Variance	18.77	-	-	-	9.33	-	-	-

* Statistically significant (*p* < 0.05).

**Table 2:** Parameter estimates for the best time series explaining abundance changes in *Culex quinquefasciatus* sampled with BG-sentinel traps at Sanmin (dengue transmission hotsop) and Nanzih (dengue transmission coldspot), Kaohsiung City, Taiwan. In parameters Intercept is the mean value of the time series and SD = Standard deviation, T = temperature, max = maximum, R = Rainfall. Lags are in biweeks.

Parameter (lag)	Sanmin				Nanzih			
	Estimates	S.E.	z	*p*-Value	Estimates	S.E.	z	*p*-Value
Intercept	142.5738	14.3782	9.92	3.55 × 10^−23^ *	63.3702	8.71	7.28	3.45 × 10^−13^ *
Tmax (1)	−22.0949	5.1298	−4.31	1.65 × 10^−5^ *	-	-	-	-
SDR (5)	-	-	-	-	1.7311	0.4884	3.54	0.00039 *
Tmax (0)	-	-	-	-	−11.8535	2.9783	−3.98	6.89 × 10^−5^ *
Error Variance	7743	-	-	-	2882	-	-	-

* Statistically significant (*p* < 0.05).

## Supplementary Materials

The following are available online at http://www.mdpi.com/2075-4450/9/3/98/s1, Figure S1: Principal Component Analysis (PCA) to assess landscape configuration, Figure S2: Seasonal boxplots of climatic variables at Kaohsiung City, Taiwan. Table S1: The characteristics of BG-Traps in the sampling area. Table S2: Model selection results for *Aedes aegypti* in Sanmin district. Table S3: Model selection results for *Aedes aegypti* in Nanzih district. Table S4: Model selection results for *Culex quinquefasciatus* in Sanmin district, Table S4: Model selection results for *Culex quinquefasciatus* in Nanzih district.

## Appendix A

Results of the Welch’s *t* test comparing the mean number of *Culex quinquefasciatus* and *Aedes aegypti* from biweeks 1 to 11 with and without the broken traps.

*Cx. quinquefasciatus* from Sanmin

Welch’s *t* = −0.7647, d.f. = 19.40, *p*-value = 0.4536

*Cx. quinquefasciatus* from Nanzih

Welch’s *t* = 0.1992, d.f. = 19.64, *p*-value = 0.8441

*Ae. aegypti* from Sanmin

Welch’s *t* = −0.1060, d.f. = 19.47, *p*-value = 0.9167

*Ae. aegypti* from Nanzih

Welch’s *t* = 0.4463, d.f. = 19.58, *p*-value = 0.6603
