# Supplementary material for: Increased Adult Aedes aegypti and Culex quinquefasciatus (Diptera: Culicidae) Abundance in a Dengue Transmission Hotspot, Compared to a Coldspot, within Kaohsiung City, Taiwan
Source: Insects. 2018 Aug 13;9(3):98. doi: 10.3390/insects9030098 (PMC6164640; doi:10.3390/insects9030098)
Supplement: Supplementary file 1 [file insects-09-00098-s001.zip › Supplementary Files/Appendix S1.pdf]

Appendix S1

Results of the Welch´s t test comparing the mean number of *Culex quinquefasciatus* and *Aedes aegypti* from biweeks 1 to 11 with and without the broken traps.

Cx. quinquefasciatus from Sanmin
Welch´s t = -0.76472, df = 19.401, p-value = 0.4536
Cx. quinquefasciatus from Nanzih
Welch´s t = 0.19923, df = 19.638, p-value = 0.8441
Ae. aegypti from Sanmin
Welch´s t = -0.10598, df = 19.473, p-value = 0.9167
Ae. aegypti from Nanzih
Welch´s t = 0.44631, df = 19.58, p-value = 0.6603
