# Supplementary material for: Increased Adult Aedes aegypti and Culex quinquefasciatus (Diptera: Culicidae) Abundance in a Dengue Transmission Hotspot, Compared to a Coldspot, within Kaohsiung City, Taiwan
Source: Insects. 2018 Aug 13;9(3):98. doi: 10.3390/insects9030098 (PMC6164640; doi:10.3390/insects9030098)
Supplement: Supplementary file 1 [file insects-09-00098-s001.zip › Supplementary Files/Figure S1.pdf]

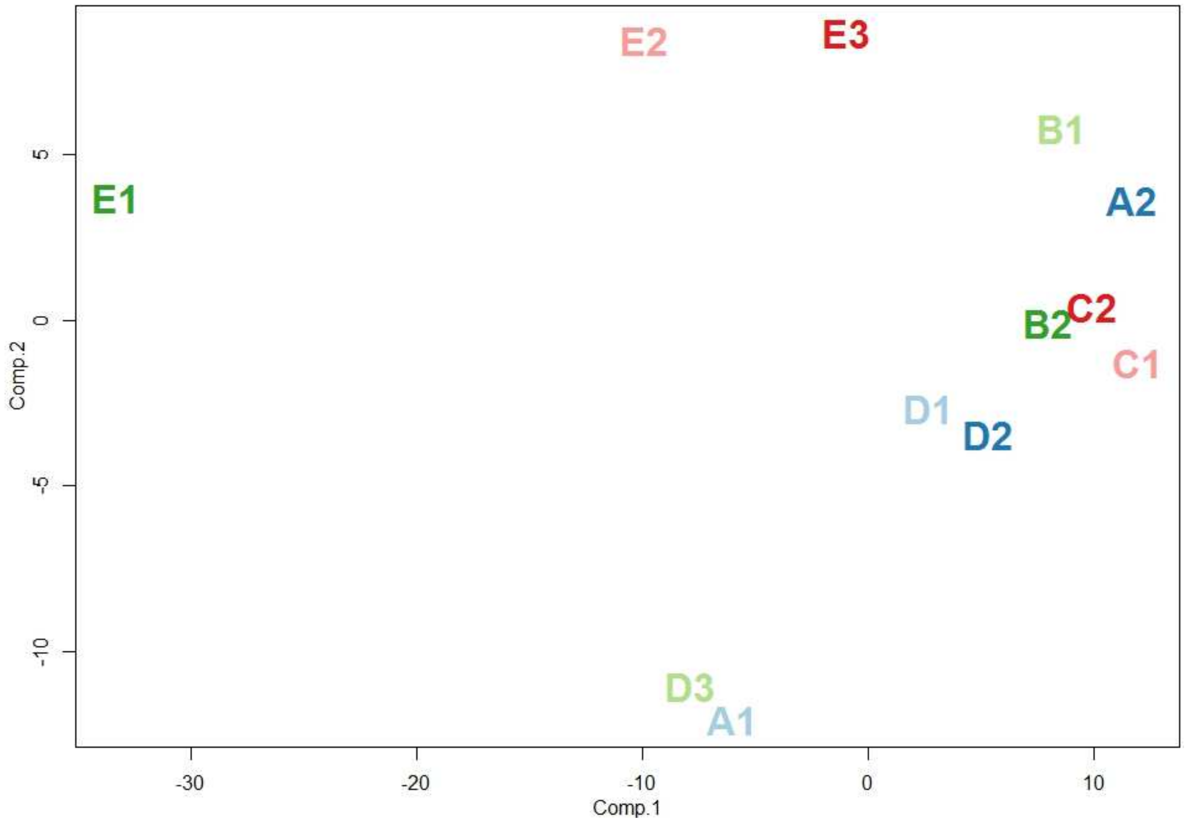

Figure S1. Principal Component Analysis (PCA) to assess landscape configuration. The x axis (Comp. 1) represents the first principal component (PC) which accounted for 74.51 % of the variability in the data. The y axis (Comp. 2) represents the second PC which accounted for 19.85 % of the variability in the data. Labels A1, A2, B1, B2, C1, C2 are for the sampling locations in Sanmin, while labels D1, D2, D3, E1, E2 and E3 are for the sampling locations in Nanzih. Label colors correspond to colors used for the different sampling locations in Figure 1 of the main text. Regarding PC interpretation, the first PC is a weighted average, whose positive values indicate a larger proportion of land devoted housing and roads when compared with recreational areas and other uses (which are dominant in locations with more negative values). Meanwhile, the second PC is more positive when more land is devoted to housing and other uses, and negative when land is devoted to roads and recreational areas. The PCA loadings used for PC interpretation is presented in a table below these lines.

Loadings	Comp.1	Comp.2	Comp.3	Comp.4
Housing	0.506	0.446	0.542	0.501
Roads	0.501	-0.348	-0.628	0.483
Recreational Areas	-0.460	-0.573	0.382	0.561
Other use	-0.530	0.593	-0.407	0.449
Cumulative Variance	0.745	0.944	0.998	1.000
