# Supplementary material for: Increased Adult Aedes aegypti and Culex quinquefasciatus (Diptera: Culicidae) Abundance in a Dengue Transmission Hotspot, Compared to a Coldspot, within Kaohsiung City, Taiwan
Source: Insects. 2018 Aug 13;9(3):98. doi: 10.3390/insects9030098 (PMC6164640; doi:10.3390/insects9030098)
Supplement: Supplementary file 1 [file insects-09-00098-s001.zip › Supplementary Files/Figure S2.pdf]

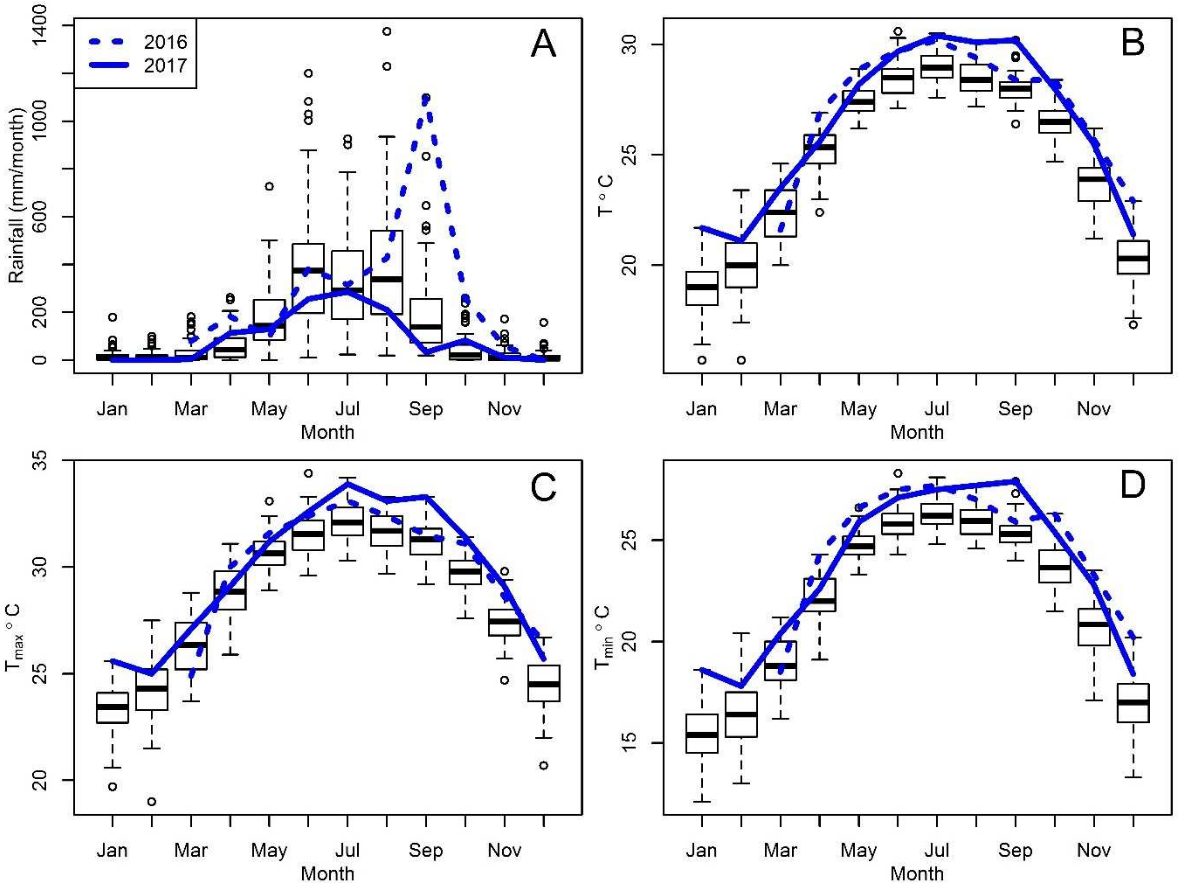

Figure S2. Seasonal boxplots of climatic variables at Kaohsiung City, Taiwan. (A) Rainfall (B) Mean Temperature (C) Maximum Temperature (D) Minimum Temperature. Boxplots were made with monthly records, from January 1960 to December 2017, collected by the Kaohsiung weather service office; ID:46744 (120.30 °E, 22.57 °N). The blue lines show the monthly estimates for the period from March 2016 to December 2017, for further details please refer to the inset legend in panel (A).
